# 3′,6′-Bis(diethyl­amino)-3*H*-spiro­[2-benzo­thio­phene-1,9′-xanthene]-3-thione

**DOI:** 10.1107/S1600536808031218

**Published:** 2008-10-04

**Authors:** Bing-Yuan Su, Xin-Qi Zhan, Jian-Nan Guo, Yue-Feng Zhou, Hong Zheng

**Affiliations:** a The Key Laboratory of Analytical Sciences, Ministry of Education, and Department of Chemistry, College of Chemistry and Chemical Engineering, Xiamen University, Xiamen 361005, People’s Republic of China; b Key Laboratory for Chemical Biology of Fujian Province, Department of Chemistry, College of Chemistry and Chemical Engineering, Xiamen University, Xiamen 361005, People’s Republic of China

## Abstract

The title compound, C_28_H_30_N_2_OS_2_, was obtained by thio­nation of 3′,6′-bis­(diethyl­amino)-3*H*-spiro­[isobenzofuran-1,9′-xan­thene]-3-one with 2,4-bis­(*p*-methoxy­phen­yl)-1,3-dithia­diphos­phetane disulfide (Lawesson’s reagent). The planes of the two benzene rings of the xanthene system are inclined at a dihedral angle of 17.4 (1)°, and the plane of the dithio­phthalide group and the planes through the two benzene rings of the xanthene system make dihedral angles of 80.2 (1) and 82.8 (1)°, respectively.

## Related literature

For related literature, see: Sun *et al.* (2008 ▶).
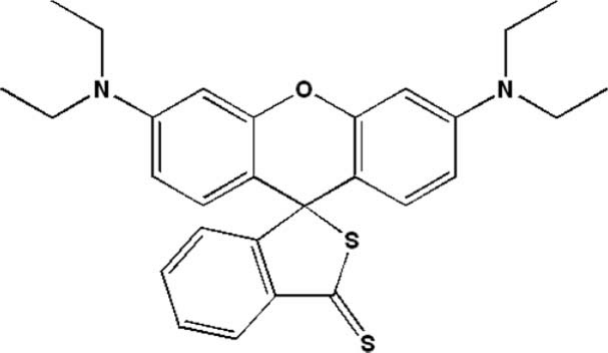


## Experimental

### 

#### Crystal data

C_28_H_30_N_2_OS_2_
*M* *_r_* = 474.66
Orthorhombic, 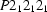

*a* = 12.181 (4) Å
*b* = 13.455 (5) Å
*c* = 15.254 (5) Å
*V* = 2500.0 (15) Å^3^
*Z* = 4
Mo *K*α radiation
μ = 0.24 mm^−1^
*T* = 173 (2) K
0.36 × 0.33 × 0.23 mm

#### Data collection

Bruker APEX area-detector diffractometer
Absorption correction: multi-scan (*SADABS*; Bruker, 2001 ▶) *T*_min_ = 0.920, *T*_max_ = 0.948
12588 measured reflections
2498 independent reflections
2114 reflections with *I* > 2σ(*I*)
*R*_int_ = 0.073

#### Refinement

*R*[*F* ^2^ > 2σ(*F* ^2^)] = 0.057
*wR*(*F* ^2^) = 0.136
*S* = 1.18
2498 reflections
298 parameters
H-atom parameters constrained
Δρ_max_ = 0.31 e Å^−3^
Δρ_min_ = −0.20 e Å^−3^

### 

Data collection: *SMART* (Bruker, 2001 ▶); cell refinement: *SAINT* (Bruker, 2001 ▶); data reduction: *SAINT*; program(s) used to solve structure: *SHELXS97* (Sheldrick, 2008 ▶); program(s) used to refine structure: *SHELXL97* (Sheldrick, 2008 ▶); molecular graphics: *ORTEP-3* (Farrugia, 1997 ▶); software used to prepare material for publication: *SHELXL97*.

## Supplementary Material

Crystal structure: contains datablocks I, global. DOI: 10.1107/S1600536808031218/kj2093sup1.cif

Structure factors: contains datablocks I. DOI: 10.1107/S1600536808031218/kj2093Isup2.hkl

Additional supplementary materials:  crystallographic information; 3D view; checkCIF report

## Figures and Tables

**Fig. 1.**
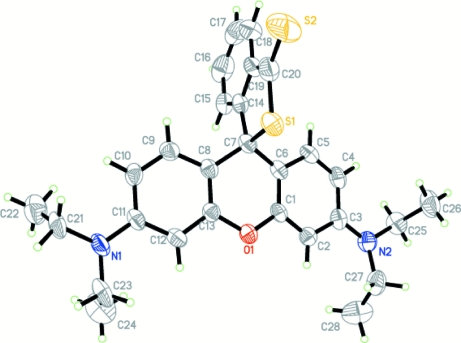
A view of the molecular structure of the title compound with the atom-labelling scheme, showing 50% probability displacement ellipsoids.

## supplementary crystallographic information

### Comment

The determination of hypochlorous acid is very important in biological systems, but it is still a challenge for the design and synthesis of highly specific and sensitive probes for hypochlorous acid (Sun *et al.*, 2008). We have therefore synthesized the title compound, and investigated its spectral responses to hypochlorous acid.

The title compound was prepared by refluxing of 3',6'-bis(diethylamino) -3*H*-spiro[isobenzofuran-1,9'-xanthene]-3-one with 2,4-di(*p*-methoxyphenyl)-1,3-dithiadiphosphetane disulfide (Lawesson's reagent) using benzene as the solvent under N_2_ atmosphere. An X-ray crystal structure determination of the molecular structure of title compound was carried out to determine its conformation. The planes of C1 / C6 and C14 / C19 rings make a dihedral angle of 80.2 (1)°, and the planes of C8 / C13 and C14 / C19 rings make a dihedral angle of 82.8 (1)°.

### Experimental

3',6'-bis(diethylamino)-3*H*-spiro[isobenzofuran-1,9'-xanthene]-3-one (1.0 g, 2.3 mmol) and 2,4-di(*p*-methoxyphenyl)-1,3-dithiadiphosphetane disulfide (1.80 g, 4.6 mmol) were dissolved in dry benzene, and the reaction mixture was refluxed for 4 h under N_2_ atmosphere. After removal of benzene, the residue was purified by flash chromatography with dichloromethane / petroleum as eluent to afford the title compound as a white solid in 26% yield. Single crystals of were obtained by slow evaporation of a dichloromethane / acetonitrile solution (20:1 *v*/*v*).

### Refinement

The hydrogen atoms were positioned geometrically (C—H = 0.93, 0.98, 0.97 or 0.96Å for phenyl, tertiary, methylene or methyl H atoms respectively) and were included in the refinement in the riding model approximation. The displacement parameters of methyl H atoms were set to 1.5*U*_eq_(C), while those of other H atoms were set to 1.2*U*_eq_(C). In the absence of significant anomalous scattering, Friedel pairs were merged.

### Crystal data

**Table:**

C_28_H_30_N_2_OS_2_	*F*(000) = 1008
*M**_r_* = 474.66	*D*_x_ = 1.261 Mg m^−^^3^
Orthorhombic, *P*2_1_2_1_2_1_	Mo *K*α radiation, λ = 0.71073 Å
Hall symbol: P 2ac 2ab	Cell parameters from 3199 reflections
*a* = 12.181 (4) Å	θ = 4.5–42.6°
*b* = 13.455 (5) Å	µ = 0.24 mm^−^^1^
*c* = 15.254 (5) Å	*T* = 173 K
*V* = 2500.0 (15) Å^3^	Block, colorless
*Z* = 4	0.36 × 0.33 × 0.23 mm

### Data collection

**Table:**

Bruker APEX area-detector diffractometer	2498 independent reflections
Radiation source: fine-focus sealed tube	2114 reflections with *I* > 2σ(*I*)
graphite	*R*_int_ = 0.073
φ and ω scans	θ_max_ = 25.0°, θ_min_ = 2.0°
Absorption correction: multi-scan (SADABS; Bruker, 2001)	*h* = −14→14
*T*_min_ = 0.920, *T*_max_ = 0.948	*k* = −15→15
12588 measured reflections	*l* = −18→13

### Refinement

**Table:**

Refinement on *F*^2^	Primary atom site location: structure-invariant direct methods
Least-squares matrix: full	Secondary atom site location: difference Fourier map
*R*[*F*^2^ > 2σ(*F*^2^)] = 0.057	Hydrogen site location: inferred from neighbouring sites
*wR*(*F*^2^) = 0.136	H-atom parameters constrained
*S* = 1.18	*w* = 1/[σ^2^(*F*_o_^2^) + (0.0662*P*)^2^] where *P* = (*F*_o_^2^ + 2*F*_c_^2^)/3
2498 reflections	(Δ/σ)_max_ = 0.003
298 parameters	Δρ_max_ = 0.31 e Å^−^^3^
0 restraints	Δρ_min_ = −0.20 e Å^−^^3^

### Special details

**Table:**

Geometry. All e.s.d.'s (except the e.s.d. in the dihedral angle between two l.s. planes) are estimated using the full covariance matrix. The cell e.s.d.'s are taken into account individually in the estimation of e.s.d.'s in distances, angles and torsion angles; correlations between e.s.d.'s in cell parameters are only used when they are defined by crystal symmetry. An approximate (isotropic) treatment of cell e.s.d.'s is used for estimating e.s.d.'s involving l.s. planes.
Refinement. Refinement of *F*^2^ against ALL reflections. The weighted *R*-factor *wR* and goodness of fit *S* are based on *F*^2^, conventional *R*-factors *R* are based on *F*, with *F* set to zero for negative *F*^2^. The threshold expression of *F*^2^ > σ(*F*^2^) is used only for calculating *R*-factors(gt) *etc*. and is not relevant to the choice of reflections for refinement. *R*-factors based on *F*^2^ are statistically about twice as large as those based on *F*, and *R*- factors based on ALL data will be even larger.

### Fractional atomic coordinates and isotropic or equivalent isotropic displacement parameters (Å^2^)

**Table:**

	*x*	*y*	*z*	*U*_iso_*/*U*_eq_	
S1	0.18097 (9)	0.90100 (7)	0.96568 (7)	0.0490 (3)	
O1	0.3456 (2)	0.65755 (17)	0.94489 (16)	0.0431 (6)	
C1	0.3709 (3)	0.7217 (2)	0.8770 (2)	0.0335 (8)	
N1	0.0836 (3)	0.4480 (2)	1.0842 (2)	0.0543 (9)	
S2	0.03347 (14)	1.07464 (9)	0.95303 (13)	0.1047 (6)	
N2	0.6222 (2)	0.7794 (2)	0.75337 (19)	0.0447 (8)	
C2	0.4781 (3)	0.7178 (2)	0.8483 (2)	0.0361 (9)	
H2	0.5268	0.6732	0.8739	0.043*	
C3	0.5144 (3)	0.7802 (2)	0.7811 (2)	0.0373 (9)	
C4	0.4354 (3)	0.8420 (3)	0.7425 (2)	0.0427 (9)	
H4	0.4551	0.8815	0.6951	0.051*	
C5	0.3288 (3)	0.8455 (2)	0.7733 (2)	0.0412 (9)	
H5	0.2793	0.8886	0.7469	0.049*	
C6	0.2932 (3)	0.7864 (2)	0.8429 (2)	0.0363 (9)	
C7	0.1800 (3)	0.7946 (2)	0.8827 (2)	0.0374 (9)	
C8	0.1525 (3)	0.7008 (2)	0.9302 (2)	0.0353 (9)	
C9	0.0457 (3)	0.6738 (2)	0.9530 (2)	0.0411 (9)	
H9	−0.0120	0.7138	0.9343	0.049*	
C10	0.0219 (3)	0.5917 (2)	1.0014 (2)	0.0426 (9)	
H10	−0.0508	0.5765	1.0140	0.051*	
C11	0.1058 (3)	0.5296 (2)	1.0326 (2)	0.0383 (9)	
C12	0.2144 (3)	0.5551 (2)	1.0098 (2)	0.0397 (9)	
H12	0.2725	0.5152	1.0281	0.048*	
C13	0.2346 (3)	0.6388 (2)	0.9605 (2)	0.0352 (8)	
C14	0.0954 (3)	0.8293 (3)	0.8183 (2)	0.0414 (9)	
C15	0.0682 (3)	0.7754 (3)	0.7464 (2)	0.0436 (10)	
H15	0.0963	0.7117	0.7387	0.052*	
C16	−0.0025 (4)	0.8164 (4)	0.6842 (3)	0.0723 (15)	
H16	−0.0217	0.7797	0.6349	0.087*	
C17	−0.0449 (4)	0.9130 (4)	0.6952 (3)	0.0732 (15)	
H17	−0.0889	0.9414	0.6522	0.088*	
C18	−0.0212 (4)	0.9629 (3)	0.7680 (4)	0.0721 (15)	
H18	−0.0519	1.0254	0.7770	0.087*	
C19	0.0501 (3)	0.9223 (3)	0.8319 (3)	0.0472 (10)	
C20	0.0808 (4)	0.9700 (3)	0.9130 (3)	0.0607 (13)	
C21	−0.0282 (3)	0.4250 (3)	1.1101 (3)	0.0532 (11)	
H21A	−0.0645	0.4864	1.1264	0.064*	
H21B	−0.0257	0.3831	1.1618	0.064*	
C22	−0.0970 (4)	0.3734 (3)	1.0412 (4)	0.0750 (15)	
H22A	−0.1692	0.3612	1.0641	0.112*	
H22B	−0.0633	0.3114	1.0256	0.112*	
H22C	−0.1022	0.4149	0.9901	0.112*	
C23	0.1720 (4)	0.3817 (3)	1.1151 (3)	0.0623 (13)	
H23A	0.2359	0.4214	1.1297	0.075*	
H23B	0.1480	0.3484	1.1682	0.075*	
C24	0.2028 (5)	0.3077 (4)	1.0508 (3)	0.0892 (18)	
H24A	0.2603	0.2667	1.0741	0.134*	
H24B	0.2283	0.3402	0.9986	0.134*	
H24C	0.1403	0.2672	1.0370	0.134*	
C25	0.6586 (4)	0.8446 (3)	0.6825 (3)	0.0505 (10)	
H25A	0.6055	0.8415	0.6351	0.061*	
H25B	0.7280	0.8203	0.6600	0.061*	
C26	0.6723 (4)	0.9514 (3)	0.7101 (3)	0.0718 (14)	
H26A	0.6962	0.9901	0.6608	0.108*	
H26B	0.7261	0.9554	0.7559	0.108*	
H26C	0.6035	0.9766	0.7310	0.108*	
C27	0.7102 (3)	0.7392 (3)	0.8084 (3)	0.0583 (12)	
H27A	0.6856	0.7392	0.8689	0.070*	
H27B	0.7732	0.7832	0.8046	0.070*	
C28	0.7463 (5)	0.6358 (4)	0.7846 (4)	0.0892 (18)	
H28A	0.8038	0.6151	0.8236	0.134*	
H28B	0.7729	0.6353	0.7254	0.134*	
H28C	0.6852	0.5912	0.7898	0.134*	

### Atomic displacement parameters (Å^2^)

**Table:**

	*U*^11^	*U*^22^	*U*^33^	*U*^12^	*U*^13^	*U*^23^
S1	0.0632 (6)	0.0381 (4)	0.0456 (5)	−0.0033 (5)	0.0015 (5)	−0.0066 (5)
O1	0.0407 (14)	0.0447 (13)	0.0439 (14)	−0.0044 (12)	0.0025 (11)	0.0169 (12)
C1	0.046 (2)	0.0284 (16)	0.0259 (16)	−0.0059 (15)	0.0015 (15)	0.0010 (15)
N1	0.057 (2)	0.0428 (17)	0.063 (2)	0.0025 (16)	0.0204 (17)	0.0246 (17)
S2	0.1242 (12)	0.0529 (7)	0.1369 (14)	0.0229 (8)	0.0171 (11)	−0.0257 (9)
N2	0.0458 (18)	0.0498 (17)	0.0383 (17)	−0.0028 (15)	0.0085 (15)	0.0050 (16)
C2	0.0407 (19)	0.0335 (17)	0.0340 (18)	−0.0021 (16)	−0.0017 (16)	−0.0037 (16)
C3	0.049 (2)	0.0358 (17)	0.0268 (17)	−0.0095 (17)	0.0015 (16)	−0.0082 (16)
C4	0.056 (2)	0.0413 (19)	0.0312 (18)	−0.0123 (18)	0.0071 (18)	0.0065 (17)
C5	0.053 (2)	0.0319 (17)	0.0388 (19)	0.0003 (17)	0.0007 (18)	0.0068 (16)
C6	0.047 (2)	0.0271 (16)	0.0353 (18)	−0.0023 (16)	0.0032 (17)	0.0005 (16)
C7	0.046 (2)	0.0283 (16)	0.0379 (18)	−0.0010 (16)	0.0036 (17)	−0.0013 (15)
C8	0.043 (2)	0.0284 (16)	0.0344 (18)	−0.0011 (15)	0.0058 (16)	−0.0031 (15)
C9	0.044 (2)	0.0348 (17)	0.045 (2)	0.0057 (16)	0.0086 (18)	0.0004 (18)
C10	0.041 (2)	0.0391 (18)	0.048 (2)	−0.0040 (18)	0.0164 (17)	−0.0032 (18)
C11	0.050 (2)	0.0271 (15)	0.0372 (18)	0.0009 (15)	0.0169 (18)	0.0004 (16)
C12	0.048 (2)	0.0334 (17)	0.038 (2)	0.0064 (16)	0.0075 (17)	0.0059 (16)
C13	0.0380 (19)	0.0349 (17)	0.0327 (18)	−0.0006 (14)	0.0064 (17)	0.0017 (17)
C14	0.042 (2)	0.0397 (18)	0.043 (2)	−0.0059 (17)	0.0066 (17)	0.0117 (18)
C15	0.046 (2)	0.046 (2)	0.039 (2)	−0.0100 (18)	0.0040 (18)	−0.0020 (19)
C16	0.064 (3)	0.104 (4)	0.050 (3)	−0.019 (3)	−0.008 (2)	0.003 (3)
C17	0.049 (3)	0.111 (4)	0.059 (3)	−0.007 (3)	−0.008 (2)	0.031 (3)
C18	0.062 (3)	0.056 (3)	0.098 (4)	0.007 (2)	0.001 (3)	0.026 (3)
C19	0.049 (2)	0.0363 (19)	0.056 (2)	−0.0017 (17)	0.007 (2)	0.0137 (19)
C20	0.057 (3)	0.047 (2)	0.079 (3)	−0.003 (2)	0.016 (2)	0.007 (2)
C21	0.067 (3)	0.043 (2)	0.050 (2)	−0.009 (2)	0.025 (2)	0.0089 (19)
C22	0.080 (3)	0.059 (3)	0.086 (3)	−0.016 (2)	0.013 (3)	−0.004 (3)
C23	0.090 (3)	0.047 (2)	0.050 (2)	−0.012 (2)	0.022 (2)	0.015 (2)
C24	0.120 (5)	0.078 (3)	0.070 (3)	0.004 (3)	0.013 (3)	−0.008 (3)
C25	0.058 (2)	0.056 (2)	0.037 (2)	−0.001 (2)	0.0161 (19)	0.0001 (19)
C26	0.073 (3)	0.057 (2)	0.086 (3)	−0.014 (2)	0.030 (3)	0.005 (3)
C27	0.053 (3)	0.071 (3)	0.051 (2)	−0.014 (2)	0.012 (2)	0.004 (2)
C28	0.088 (4)	0.099 (4)	0.081 (4)	0.027 (3)	−0.011 (3)	−0.008 (3)

### Geometric parameters (Å, °)

**Table:**

S1—C20	1.732 (5)	C15—C16	1.395 (6)
S1—C7	1.911 (3)	C15—H15	0.9300
O1—C1	1.383 (4)	C16—C17	1.408 (7)
O1—C13	1.396 (4)	C16—H16	0.9300
C1—C2	1.379 (5)	C17—C18	1.329 (7)
C1—C6	1.388 (5)	C17—H17	0.9300
N1—C11	1.378 (4)	C18—C19	1.415 (6)
N1—C21	1.452 (5)	C18—H18	0.9300
N1—C23	1.476 (6)	C19—C20	1.442 (6)
S2—C20	1.639 (4)	C21—C22	1.513 (6)
N2—C3	1.380 (5)	C21—H21A	0.9700
N2—C25	1.461 (5)	C21—H21B	0.9700
N2—C27	1.465 (5)	C22—H22A	0.9600
C2—C3	1.397 (5)	C22—H22B	0.9600
C2—H2	0.9300	C22—H22C	0.9600
C3—C4	1.401 (5)	C23—C24	1.448 (6)
C4—C5	1.381 (5)	C23—H23A	0.9700
C4—H4	0.9300	C23—H23B	0.9700
C5—C6	1.395 (5)	C24—H24A	0.9600
C5—H5	0.9300	C24—H24B	0.9600
C6—C7	1.510 (5)	C24—H24C	0.9600
C7—C8	1.493 (4)	C25—C26	1.506 (6)
C7—C14	1.499 (5)	C25—H25A	0.9700
C8—C13	1.383 (5)	C25—H25B	0.9700
C8—C9	1.395 (5)	C26—H26A	0.9600
C9—C10	1.360 (5)	C26—H26B	0.9600
C9—H9	0.9300	C26—H26C	0.9600
C10—C11	1.403 (5)	C27—C28	1.503 (6)
C10—H10	0.9300	C27—H27A	0.9700
C11—C12	1.409 (5)	C27—H27B	0.9700
C12—C13	1.376 (5)	C28—H28A	0.9600
C12—H12	0.9300	C28—H28B	0.9600
C14—C15	1.355 (5)	C28—H28C	0.9600
C14—C19	1.384 (5)		
			
C20—S1—C7	95.16 (19)	C18—C17—H17	120.5
C1—O1—C13	117.2 (3)	C16—C17—H17	120.5
C2—C1—O1	115.2 (3)	C17—C18—C19	120.9 (4)
C2—C1—C6	123.5 (3)	C17—C18—H18	119.5
O1—C1—C6	121.3 (3)	C19—C18—H18	119.5
C11—N1—C21	120.7 (3)	C14—C19—C18	119.4 (4)
C11—N1—C23	121.4 (3)	C14—C19—C20	115.3 (4)
C21—N1—C23	117.9 (3)	C18—C19—C20	125.3 (4)
C3—N2—C25	120.7 (3)	C19—C20—S2	127.6 (4)
C3—N2—C27	121.6 (3)	C19—C20—S1	110.0 (3)
C25—N2—C27	115.1 (3)	S2—C20—S1	122.4 (3)
C1—C2—C3	120.7 (3)	N1—C21—C22	115.4 (4)
C1—C2—H2	119.7	N1—C21—H21A	108.4
C3—C2—H2	119.7	C22—C21—H21A	108.4
N2—C3—C2	121.4 (3)	N1—C21—H21B	108.4
N2—C3—C4	122.0 (3)	C22—C21—H21B	108.4
C2—C3—C4	116.6 (3)	H21A—C21—H21B	107.5
C5—C4—C3	121.5 (3)	C21—C22—H22A	109.5
C5—C4—H4	119.2	C21—C22—H22B	109.5
C3—C4—H4	119.2	H22A—C22—H22B	109.5
C4—C5—C6	122.2 (3)	C21—C22—H22C	109.5
C4—C5—H5	118.9	H22A—C22—H22C	109.5
C6—C5—H5	118.9	H22B—C22—H22C	109.5
C1—C6—C5	115.5 (3)	C24—C23—N1	112.8 (4)
C1—C6—C7	121.2 (3)	C24—C23—H23A	109.0
C5—C6—C7	123.3 (3)	N1—C23—H23A	109.0
C8—C7—C14	115.3 (3)	C24—C23—H23B	109.0
C8—C7—C6	109.8 (3)	N1—C23—H23B	109.0
C14—C7—C6	112.7 (3)	H23A—C23—H23B	107.8
C8—C7—S1	108.3 (2)	C23—C24—H24A	109.5
C14—C7—S1	101.8 (2)	C23—C24—H24B	109.5
C6—C7—S1	108.4 (2)	H24A—C24—H24B	109.5
C13—C8—C9	115.7 (3)	C23—C24—H24C	109.5
C13—C8—C7	120.7 (3)	H24A—C24—H24C	109.5
C9—C8—C7	123.4 (3)	H24B—C24—H24C	109.5
C10—C9—C8	123.0 (3)	N2—C25—C26	113.5 (3)
C10—C9—H9	118.5	N2—C25—H25A	108.9
C8—C9—H9	118.5	C26—C25—H25A	108.9
C9—C10—C11	120.9 (3)	N2—C25—H25B	108.9
C9—C10—H10	119.6	C26—C25—H25B	108.9
C11—C10—H10	119.6	H25A—C25—H25B	107.7
N1—C11—C10	121.7 (3)	C25—C26—H26A	109.5
N1—C11—C12	121.2 (3)	C25—C26—H26B	109.5
C10—C11—C12	117.1 (3)	H26A—C26—H26B	109.5
C13—C12—C11	120.1 (3)	C25—C26—H26C	109.5
C13—C12—H12	120.0	H26A—C26—H26C	109.5
C11—C12—H12	120.0	H26B—C26—H26C	109.5
C12—C13—C8	123.2 (3)	N2—C27—C28	114.7 (4)
C12—C13—O1	114.5 (3)	N2—C27—H27A	108.6
C8—C13—O1	122.3 (3)	C28—C27—H27A	108.6
C15—C14—C19	120.5 (4)	N2—C27—H27B	108.6
C15—C14—C7	122.1 (3)	C28—C27—H27B	108.6
C19—C14—C7	117.2 (3)	H27A—C27—H27B	107.6
C14—C15—C16	119.3 (4)	C27—C28—H28A	109.5
C14—C15—H15	120.4	C27—C28—H28B	109.5
C16—C15—H15	120.4	H28A—C28—H28B	109.5
C15—C16—C17	120.7 (4)	C27—C28—H28C	109.5
C15—C16—H16	119.7	H28A—C28—H28C	109.5
C17—C16—H16	119.7	H28B—C28—H28C	109.5
C18—C17—C16	119.1 (5)		
			
C13—O1—C1—C2	−163.2 (3)	C9—C10—C11—C12	−1.5 (5)
C13—O1—C1—C6	18.4 (4)	N1—C11—C12—C13	−177.9 (3)
O1—C1—C2—C3	−178.9 (3)	C10—C11—C12—C13	1.3 (5)
C6—C1—C2—C3	−0.5 (5)	C11—C12—C13—C8	−0.8 (5)
C25—N2—C3—C2	179.5 (3)	C11—C12—C13—O1	178.0 (3)
C27—N2—C3—C2	−19.7 (5)	C9—C8—C13—C12	0.4 (5)
C25—N2—C3—C4	0.6 (5)	C7—C8—C13—C12	175.4 (3)
C27—N2—C3—C4	161.4 (3)	C9—C8—C13—O1	−178.3 (3)
C1—C2—C3—N2	178.0 (3)	C7—C8—C13—O1	−3.3 (5)
C1—C2—C3—C4	−3.1 (5)	C1—O1—C13—C12	162.1 (3)
N2—C3—C4—C5	−177.0 (3)	C1—O1—C13—C8	−19.1 (5)
C2—C3—C4—C5	4.1 (5)	C8—C7—C14—C15	−63.5 (4)
C3—C4—C5—C6	−1.5 (5)	C6—C7—C14—C15	63.7 (4)
C2—C1—C6—C5	3.1 (5)	S1—C7—C14—C15	179.5 (3)
O1—C1—C6—C5	−178.7 (3)	C8—C7—C14—C19	120.4 (4)
C2—C1—C6—C7	−173.9 (3)	C6—C7—C14—C19	−112.4 (4)
O1—C1—C6—C7	4.3 (5)	S1—C7—C14—C19	3.5 (4)
C4—C5—C6—C1	−2.0 (5)	C19—C14—C15—C16	2.7 (6)
C4—C5—C6—C7	174.9 (3)	C7—C14—C15—C16	−173.2 (4)
C1—C6—C7—C8	−24.2 (4)	C14—C15—C16—C17	0.4 (6)
C5—C6—C7—C8	159.0 (3)	C15—C16—C17—C18	−3.3 (7)
C1—C6—C7—C14	−154.2 (3)	C16—C17—C18—C19	3.2 (7)
C5—C6—C7—C14	29.0 (4)	C15—C14—C19—C18	−2.9 (6)
C1—C6—C7—S1	93.9 (3)	C7—C14—C19—C18	173.2 (4)
C5—C6—C7—S1	−82.9 (4)	C15—C14—C19—C20	176.8 (3)
C20—S1—C7—C8	−121.4 (3)	C7—C14—C19—C20	−7.0 (5)
C20—S1—C7—C14	0.6 (3)	C17—C18—C19—C14	−0.1 (7)
C20—S1—C7—C6	119.6 (3)	C17—C18—C19—C20	−179.8 (4)
C14—C7—C8—C13	152.1 (3)	C14—C19—C20—S2	−173.9 (3)
C6—C7—C8—C13	23.5 (4)	C18—C19—C20—S2	5.8 (6)
S1—C7—C8—C13	−94.6 (3)	C14—C19—C20—S1	7.0 (5)
C14—C7—C8—C9	−33.2 (5)	C18—C19—C20—S1	−173.3 (4)
C6—C7—C8—C9	−161.8 (3)	C7—S1—C20—C19	−4.1 (3)
S1—C7—C8—C9	80.0 (4)	C7—S1—C20—S2	176.8 (3)
C13—C8—C9—C10	−0.5 (5)	C11—N1—C21—C22	79.6 (5)
C7—C8—C9—C10	−175.4 (3)	C23—N1—C21—C22	−100.7 (4)
C8—C9—C10—C11	1.1 (5)	C11—N1—C23—C24	−83.3 (5)
C21—N1—C11—C10	−1.7 (5)	C21—N1—C23—C24	97.0 (5)
C23—N1—C11—C10	178.6 (3)	C3—N2—C25—C26	76.0 (5)
C21—N1—C11—C12	177.5 (4)	C27—N2—C25—C26	−85.9 (4)
C23—N1—C11—C12	−2.2 (5)	C3—N2—C27—C28	100.4 (4)
C9—C10—C11—N1	177.8 (3)	C25—N2—C27—C28	−97.8 (4)

### Hydrogen-bond geometry (Å, °)

**Table:**

*D*—H···*A*	*D*—H	H···*A*	*D*···*A*	*D*—H···*A*
C17—H17···Cg^i^	0.93	3.14	3.961 (5)	149

Symmetry codes: (i) −*x*, *y*+1/2, −*z*+3/2.
